# Characterization of Ultrasonic Energy Diffusion in a Steel Alloy Sample with Tensile Force Using PZT Transducers

**DOI:** 10.3390/s19092185

**Published:** 2019-05-11

**Authors:** Guangtao Lu, Tao Wang, Mingle Zhou, Yourong Li

**Affiliations:** 1 Key Laboratory for Metallurgical Equipment and Control of Ministry of Education, Wuhan University of Science and Technology, Wuhan 430081, China; 13275828822@163.com; 2 Hubei Key Laboratory of Mechanical Transmission and Manufacturing Engineering, Wuhan University of Science and Technology, Wuhan 430081, China; wangtao77@wust.edu.cn (T.W.); liyourong@wust.edu.cn (Y.L.)

**Keywords:** piezoceramic transducers, ultrasonic energy diffusion, tensile force, deformation, ultrasound, force identification, structural health monitoring

## Abstract

During the propagation of ultrasound in a polycrystalline material, ultrasonic energy losses due to the scattering at the boundaries between grains is usually described by the ultrasonic energy diffusion equation, and the boundaries of the grains in the material are influenced by the structural load. The aim of this research is to investigate the characterization of ultrasonic energy diffusion in a steel alloy sample under structural load by using lead zirconate titanate (PZT) transducers. To investigate the influence of structural load on ultrasonic energy diffusion, an experimental setup of a steel alloy plate under different tensile forces is designed and four samples with similar dimensions are fabricated. The experimental results of the four samples reveal that, during the loading process, the normalized ultrasonic energy diffusion coefficient fluctuates firstly, then decreases and at last increases as the tensile force increases. The proposed tensile force index shows a similar changing trend to the recorded displacement of the sample. Moreover, when the tensile force is less than the lower yield point or the sample deforms elastically, the index can be approximated by a cubic model. Therefore, the proposed tensile force index can be used to monitor the tensile force in the elastic deformation stage. Moreover, based on these findings, some force evaluation methods and their potential applications, such as the preloading detection of bolts, can be developed based on the linear relationships between the proposed index and the applied force.

## 1. Introduction

Structural health monitoring (SHM) systems have made much progress in improving the reliability and safety of structures and reducing the operating and maintenance cost in recent years [1,2,3]. In SHM systems, ultrasonic waves are often used and PZT transducers, due to their strong piezoelectric effect and high bandwidth [4,5], are often employed to generate and detect these waves in structures [6,7,8,9,10,11]. The detected waves are then further processed to estimate the operating parameters and health state of the structure [12,13,14], such as the stiffness change of a material [15,16,17], the looseness of bolts [18,19], and the curing state of different materials [20,21], or to identify structural damages, including delamination [22,23,24], cracking [25,26], and corrosion [27].

In fact, for structural damage, such as fatigue cracks [28], corrosion cracks [29], debonding [30,31] and delamination, the structural loads play an important role [32]. Lim and Soh [33,34] investigated the characterization of a propagating crack induced by varying axial load, and the crack was monitored by using electromechanical impedance (EMI) technique. Once damages occur, they consequently change the propagating characteristics, especially the energy, of the ultrasound [35,36]. Recently, the influence of damage on the ultrasonic energy in such situations has been investigated by many researchers, and some ultrasonic-energy-based methods using PZTs are developed to quantitatively estimate the size of this damage [37,38]. Mi et al. studied the behavior of the received energy under different loads, and proposed a new method for crack detection based on the relationship between the received energy and the load [39]. Inn and Chang employed an ultrasonic-energy-based index to quantitatively measure the growth of a simulated fatigue crack by using a built-in PZT network [40]. Feng et al. investigated the debonding characteristics in concrete by applying tensile force to the structure, and an ultrasonic energy index was used to detect the debonding between the two rubber–steel layers [41]. Wang et al. monitored the looseness of bolts by detecting the preload of the bolts, and a series of experiments showed that the stiffness of the electromechanical coupling system was affected by the preload [42]. However, these studies mainly revolve around how to make use of the ultrasonic energy loss to detect a damage, and the scattering characteristics of ultrasonic waves in a damaged medium is paid little attention.

Recently, some efforts have been made to investigate the mechanism of ultrasonic energy losses in a medium from the microscopic point of view. Some studies [43,44,45] pointed out that ultrasonic waves are attenuated by scattering at boundary between the grains during the propagation in a polycrystalline material. The ultrasonic energy attenuation or losses mainly includes ultrasonic energy diffusion and energy dissipation. These studies also indicate that the ultrasonic energy dissipation is mainly related with its viscoelastic characteristics of the medium while the ultrasonic energy diffusion is mainly affected by the microstructures, such as the grain and micro-damages distributed in the medium [43,44,45]. Weaver [43,44] derived an ultrasonic diffusion equation to mathematically investigate the energy scattering in polycrystals with microstructure. His study indicated that the ultrasonic energy diffusivity was influenced by some parameters, such as the micro-scale length and material Poisson ratio, of the microstructures. A much simpler expression was obtained in media with polydispersed scatterers by Turner et al. [46], and this expression showed that the ultrasonic energy diffusivity was a function of the volume fraction of the scatterers in the medium. To validate these findings, experimental and numerical studies were conducted by Anugonda et al. [47,48] and Schubert and Koehler [49], respectively. Since the ultrasonic scattering characterizations of cracks, especially micro-cracks, is quite similar to the scatterers or aggregates distributed in concrete, cracks show a similar influence on ultrasonic energy diffusion. Therefore, a damage detection based on ultrasonic energy diffusion was developed by researchers [50,51,52,53] to identify cracks in concrete structures. Their experimental results indicated that the ultrasonic energy diffusivity was indeed related to the size of the crack. Recently, this method was further developed to detect small-size hole-like damage in an aluminum plate by PZT transducers [54]. These studies in concrete and aluminum structures show promise for structural micro-damage or initial damage detection based on ultrasonic energy diffusion.

Moreover, since the occurrence and growth of structural damage is usually associated with structural load, there may be a certain connection between the structural load and ultrasonic energy losses or ultrasonic energy diffusion, and this relationship has potential for structural load measurement or load-involved damage detection. However, previous investigations only focus on how the microstructures including the damage distributed in the medium influence ultrasonic energy losses or ultrasonic energy diffusion, and how the structural load influences ultrasonic energy diffusion is paid little attention. Therefore, the characteristics of ultrasonic energy diffusion in a steel alloy plate with tensile force are investigated with the help of PZT transducers. We hope that the findings in this study can offer guidelines for future development of force evaluation based on ultrasonic energy diffusion.

The rest of the paper is organized as following. In Section 2, the theory of ultrasonic energy diffusion in polycrystalline materials is presented and the process of determining some parameters of ultrasonic energy diffusion is described. In Section 3, an experimental setup of a steel alloy plate in the universal material test machine is designed to investigate the characterizations of ultrasound energy diffusion under tensile force. The experimental results are analyzed and discussed in Section 4. Finally, Section 5 concludes the paper.

## 2. Theory of Ultrasonic Energy Diffusion in Polycrystalline Materials

Since Mason and McSkimin [55] demonstrated that an elastic wave scatters and attenuates in microstructures when it travels in a polycrystalline medium, some consistent efforts have been made to quantitatively evaluate the energy losses and to advance its applications in the field of NDE (non-destructive evaluation) and SHM.

Weaver and his collaborator developed a theory of ultrasonic energy diffusion to characterize the energy scattering at the microstructures in polycrystalline materials. In this theory, the ultrasonic energy attenuated at the scatterers is described by the spectral energy density [56], and the spatio-temporal evolution of spectral energy density $E(\vec{r},t)$ at point $\vec{r}$ at time *t* is given by

(1) $$\frac{\partial E(\vec{r},t)}{\partial t}+D\nabla^{2}E(\vec{r},t)-\sigma E(\vec{r},t)=E_{0}\delta (\vec{r}-{\vec{r}}_{0})\delta (t-t_{0})$$

where *E*_0_ is the initial energy of the transmitted pulse at point ${\vec{r}}_{0}$ at time *t*_0_, *D* is the ultrasonic energy diffusion coefficient, *σ* is the ultrasonic energy dissipation coefficient, and *δ* is the Dirac delta-function.

In Equation (1), the parameter *σ* is mainly determined by the viscoelastic characteristics of the medium and this parameter is usually associated with energy absorption by the medium itself. The parameter *D* is mainly influenced by the microstructures distributed in the medium, where the microstructures include the randomly distributed grains and the micro-damages generated in the medium. Therefore, the ultrasonic energy diffusion coefficient *D* can be taken as an index of the change of the microstructure.

On the other hand, when a force is applied onto a material, the material deforms macroscopically, and the shape and boundaries of the grains distributed in the medium change microscopically. Moreover, these effects become more and more obvious as the force increases. During this process, the spectral energy density and ultrasonic energy diffusion coefficient *D* consequently change. Therefore, the spectral energy density and ultrasonic energy diffusion can be employed to reflect the change of the microstructure in the medium and the applied force on the structure.

To simplify Equation (1), a one-dimensional structure is considered in this paper and the spectral energy density in Equation (1) is given by

(2) $$E(x,t)=E_{0}\frac{1}{2\sqrt{\pi Dt}}e^{-\frac{x^{2}}{4Dt}}e^{-\sigma t}$$

where x is the distance between the two points where the pulse is transmitted and received.

Equation (2) indicates that the spectral energy density increases firstly and then decays exponentially. Equation (2) is re-formatted in the logarithmic form as

(3) $$\ln E(x,t)=C_{0}-\frac{x^{2}}{4Dt}-\sigma t-0.5\ln(Dt)$$

where *C*_0_ is a constant that depends on the initial energy.

For a given structure, the spectral energy density in Equation (3) is computed by the raw temporal signals without pre-processing and then the three parameters *C*_0_, *D*, and *σ* are obtained by curve fitting through the following four steps [54]:

1. Divide the recorded temporal signal into short segments with a window length *Δt* and a window overlap ratio *γ*, and the window overlap ratio *γ* is usually set to be 0.9;
2. Determine the power spectrum of each individual temporal segment by discrete time Fourier transform (DTFT);
3. Compute the ultrasonic energy density of each individual temporal segment by adding up the power spectrum in a frequency bandwidth *Δf* with a center frequency *f_c_*;
4. Finaly, obtain the ultrasound energy diffusion coefficients in Equation (3) just by curve fitting.

## 3. Experimental Setup

**Figure 1:**
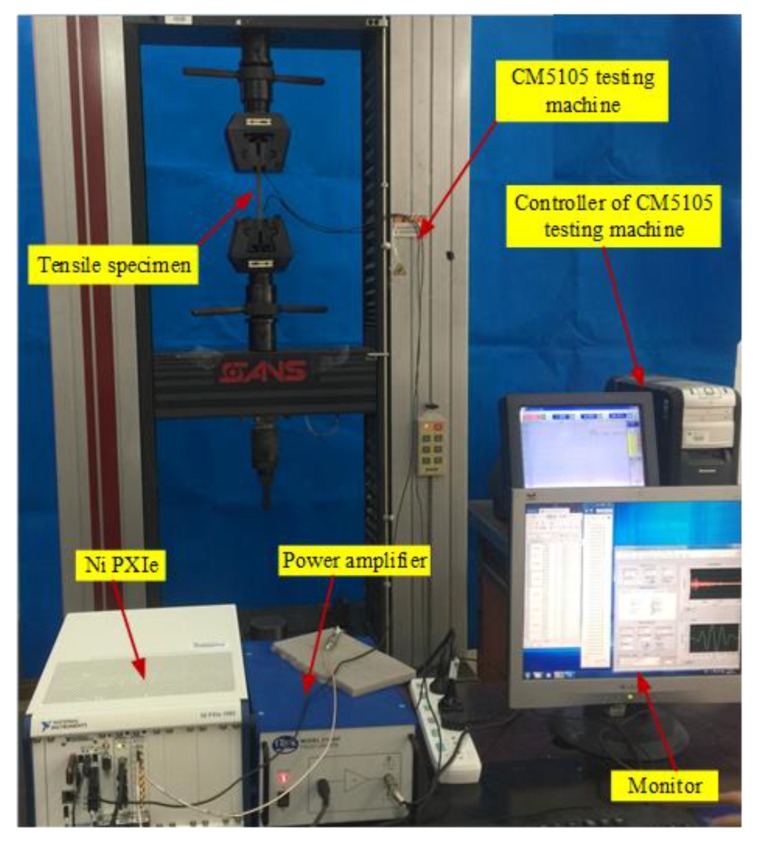
Experimental setup for the data acquisition system.

**Figure 2:**
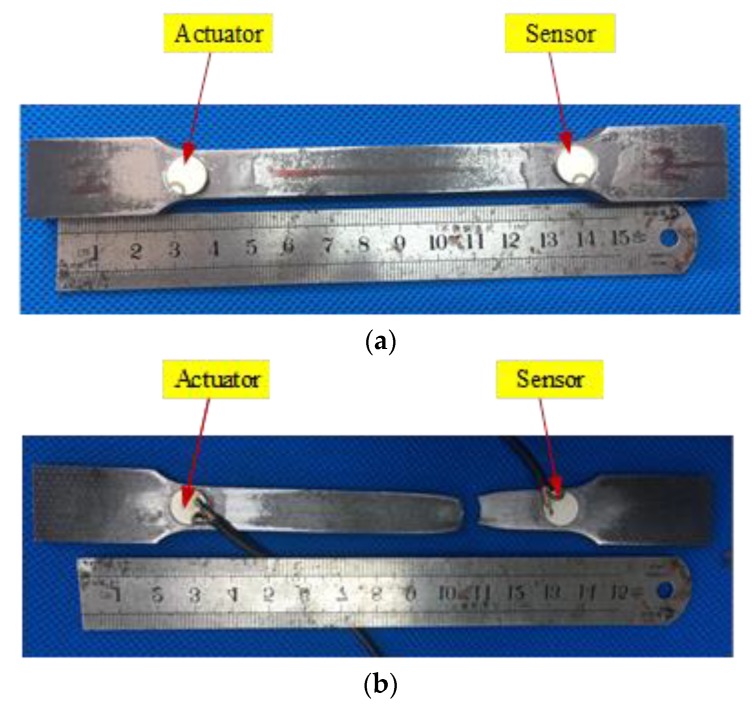
Tensile samples with two PZTs: (**a**) Before the test; (**b**) After the destructive tests.

To verify the performance of the ultrasonic energy diffusion based tensile force identification method, an experimental setup is designed, as shown in Figure 1. The experimental setup mainly consists of an universal material testing machine with a maximum load of 100 kN (Model CMT5105, SUNS), a tensile sample (material type: GB/T 70-1988 Q235 which is equivalent to ASTM A36) bonded with two PZTs by epoxy (shown in Figure 2), a power amplifier with a 50 times voltage gain and a bandwidth 0~2.6 MHz for piezoceramic actuation (Trek Model 2100H), a signal generation and data acquisition system (an Ni PXIe-1082 chassis with an Ni PXIe-5243 arbitrary waveform generator and an Ni PXIe-5105 digitizer) and a monitor.

**Figure 3:**
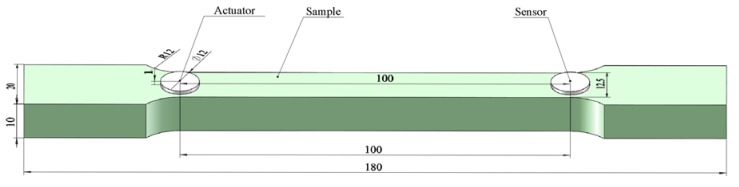
Dimensions of the sample and the PZTs (unit: mm).

The tensile sample is designed and fabricated according to the standard of GB/T 228-2002 [57] which is equal to the standard of ISO 6892: 1998 [58]. The width of the parallel length is selected from the standard, and the other dimensions are designed for consideration of the fixture of the testing machine. The dimensions of the sample are shown as in Figure 3. As shown in Figure 2, two PZT disks with a dimension Φ12 × 1 mm are bonded on the sample by epoxy (Deli Epoxy Resin 7148 from Deli Group Co., Ltd., Shanghai, China). To avoid the PZT transducers at the breaking point, the two PZT transducers are placed near to the two ends of the sample and the positions of these two PZT transducers are plotted in Figure 3. To decrease the adverse effect of the thickness and property of the epoxy, the PZT transducers are bonded in three steps as below: (1) before the bonding, two optical fibers with a diameter of 0.125 mm are placed on the bonding area to control the thickness of the epoxy and the epoxy is applied evenly on this area; (2) the PZT transducer is fixed and pressed by the same pressure for at least 5 minutes firstly; and (3) they are kept at room temperature for at least 24 h to make sure the epoxy is fully cured. In the test, one of these two PZTs is selected as an actuator to transmit a pulse actively, and the other one is employed as a sensor to detect the waves, as shown in Figure 3.

**Figure 4:**
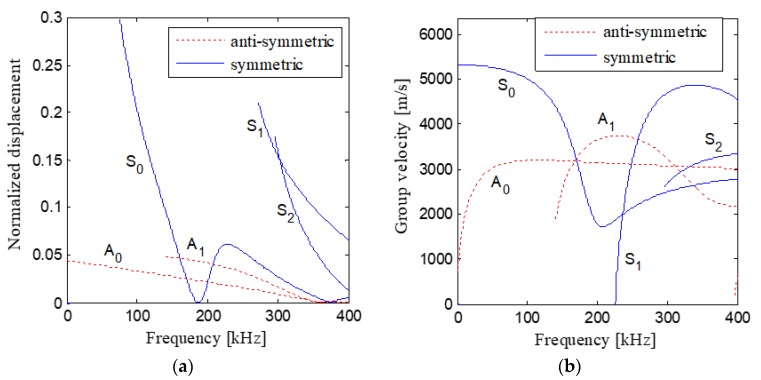
Normalized displacement and group velocity of different modes of Lamb wave excited in the sample: (**a**) Normalized displacement; (**b**) Group velocity.

**Figure 5:**
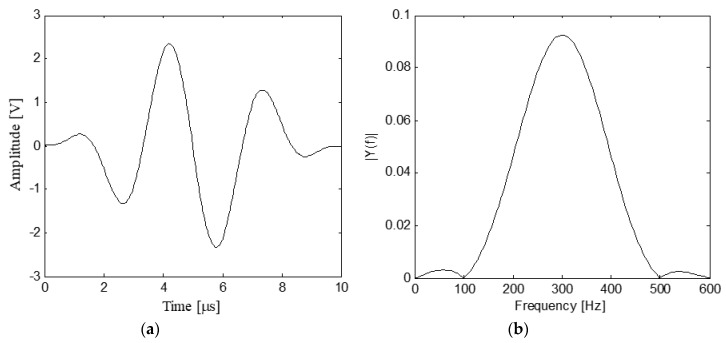
Curves of the excitation pulse in time and frequency domains: (**a**) Time domain; (**b**) Frequency domain.

As shown in Figure 4, when the excitation frequency is about 300 kHz, both the normalized displacement and group velocity of S_1_ mode are much larger than other modes’, and therefore, S_1_ mode is the dominant mode at this frequency and it transports most of the energy of the waves [54,59]. Since the energy of a Hanning-windowed tone burst mainly focuses at the center frequency of the pulse, the Hanning-windowed tone burst is selected in the experiments [60]. Moreover, the output of the power amplifier with 50 times voltage gain (Trek Model 2100H) is required to be less than ±150 V. Therefore, the Hanning-windowed tone burst with a peak amplitude of 2.5 volt and 3 peaks is selected in the experiments, and the center frequency of the excitation pulse is set to be 300 kHz. Figure 5 shows the excitation pulse in time and frequency domains.

Since the yield stress of Q235 is 235 MPa, the yield force of the sample is 235 MPa × 12.5 mm × 10 mm = 29.4 kN. Similarly, the tensile strength of Q235 is 370~460 MPa and the maximum tensile force that the sample yields is 46.3~57.5 kN. Therefore, the tensile force is applied to the sample by 10 steps in the elastic deformation stage. During the whole experiment, the sample is applied with a tensile force from 0 kN with a load step of 3 kN by the universal material testing machine until the sample breaks down. During each load step, the tone burst is firstly generated by the Ni PXIe-5243 arbitrary waveform generator at a sampling frequency of 20 MHz, then amplified and sent to the actuator. The ultrasonic wave is detected by the PZT sensor and recorded by the Ni PXIe-5105 digitizer at a sampling frequency of 60 MHz. In the trial tests, the amplitude of the waves decays to zero when the time is 0.6 ms, and therefore the total recording time is selected to be 0.6 ms in the whole test.

In the experiments, four samples with similar dimensions (Samples L1~L4) were tested. Among these four samples, three samples with two PZTs (Samples L1~L3) are tested to investigate the characteristics of ultrasonic energy diffusion with different loads. Sample L4 is employed to obtain the deformation curve and the maximum tensile force which these samples can yield.

## 4. Experimental Results

### 4.1. Ultrasonic Energy Density

**Figure 6:**
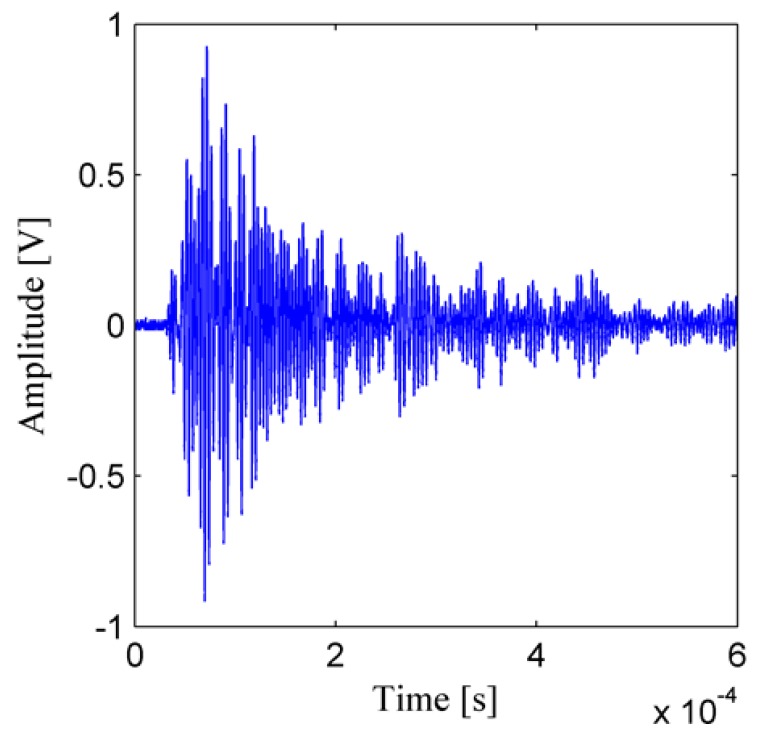
Response of the structure to the excitation pulse.

**Figure 7:**
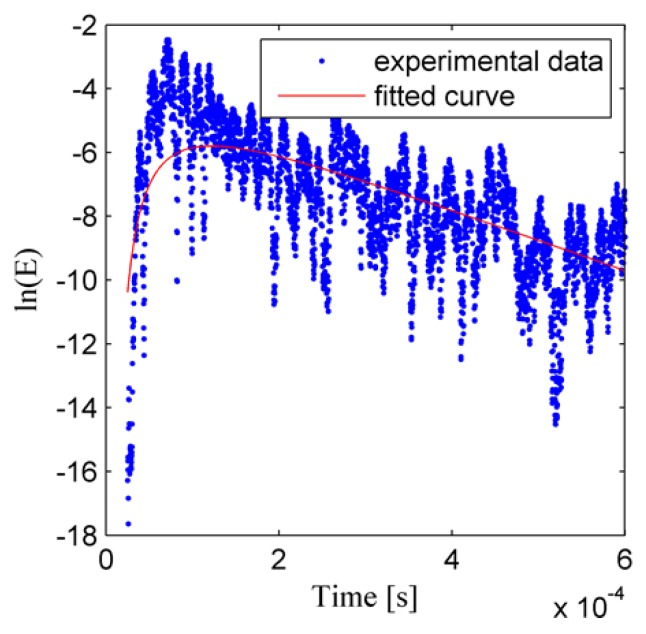
Ultrasonic energy density curve with a tensile force 48 kN.

After the recorded temporal signals are processed by the four steps, the ultrasonic energy density and the two ultrasonic energy diffusion coefficients are obtained. Figure 6 shows the temporal signal which is the response of the structure to the excitation pulse. To obtain a good resolution and a small fluctuation of the ultrasonic energy density [46,48,51,54], some parameters are chosen as time window length Δ*t* = 1.67 μs, time window overlap ratio *γ* = 0.9, frequency bandwidth Δ*f* = 30 kHz, frequency center *f_c_* = 300 kHz, and all the recorded signals are processed as described in Section 2. The ultrasonic energy density curve with a tensile force 48 kN is plotted in Figure 7, and the red curves in Figure 7 are obtained by curve fitting. As shown in Figure 7, due to the fluctuation of the ultrasonic energy density itself [43,44,46], there is an error between the fitted curve and the computed ultrasonic energy density.

**Figure 8:**
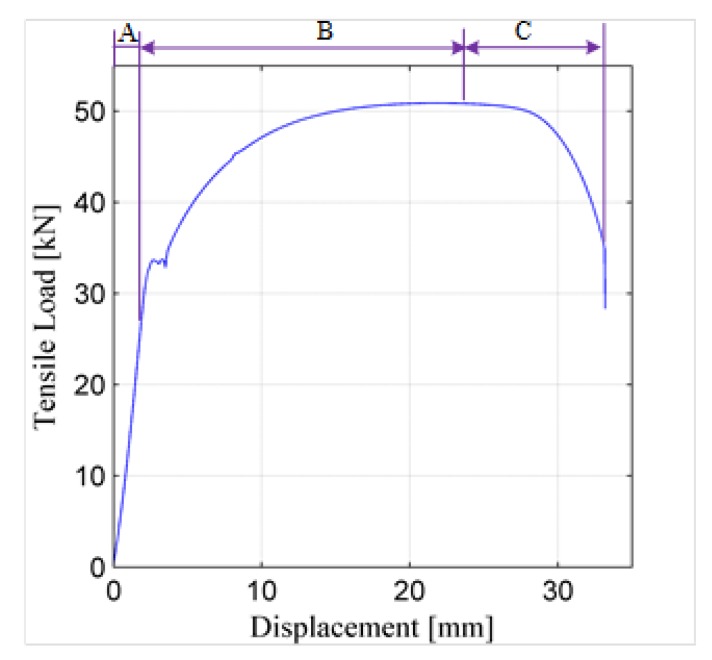
Deformation curve of sample L4 under tensile force. (A—Elastic deformation stage; B—Plastic deformation stage; C—Necking stage).

During the test of Sample L4, some key parameters—including the deformation, tensile force, and lower and the upper yield points—are measured by the test machine. Figure 8 shows the deformation curve of Sample L4, and the measured lower and upper yield points are 29.3 kN and 33.65 kN, respectively. As shown in Figure 8, the whole deformation process includes three stages: the elastic deformation stage, plastic deformation stage, and necking stage. In the elastic deformation stage where the tensile load is less than the lower yield point, the deformation displacement increases linearly to the tensile load. In the plastic deformation stage, the deformation displacement increases nonlinearly to the load until the load reaches the peak point. In the necking stage, the displacement still increases while the load decreases, and the sample breaks down quickly.

### 4.2. Influence of Tensile Force on Ultrasonic Energy Diffusion

To decrease the adverse effect of the location error of the PZTs and the variation of the clamping force at the ends of the sample, the ultrasonic energy diffusion coefficients under different tensile loads are normalized by the value when there is no tensile force applied on the sample.

**Figure 9:**
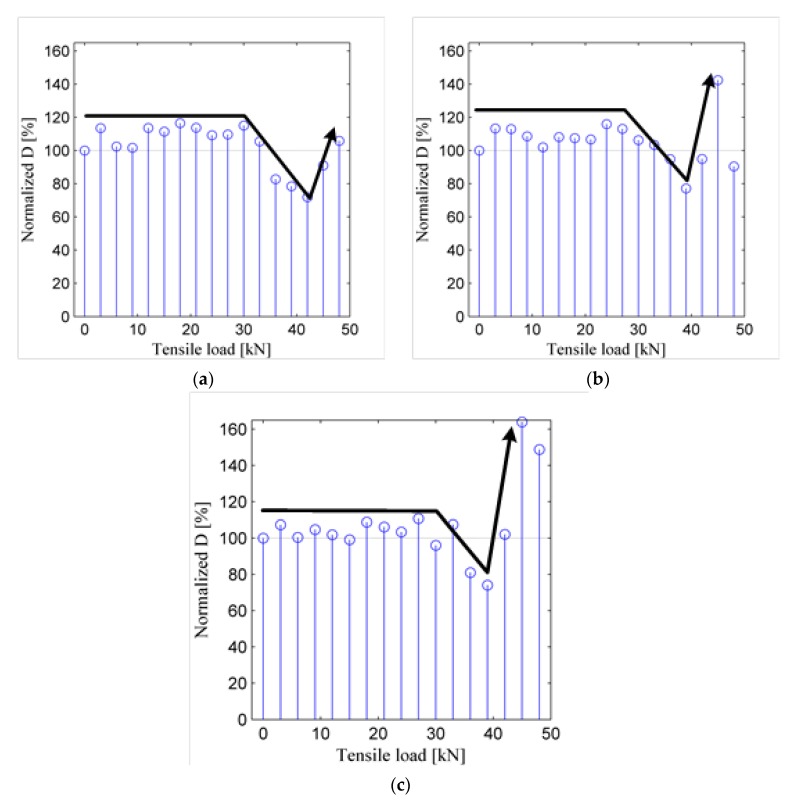
Normalized ultrasonic energy coefficients of three samples under tensile forces: (**a**) Sample L1; (**b**) Sample L2; (**c**) Sample L3.

Figure 9 plots the normalized ultrasonic energy coefficients versus the tensile force for the three samples. Figure 9 demonstrates that the normalized energy diffusion coefficients of the three samples show a similar changing trend as the tensile force increases. When the tensile force is less than 30 kN, which is near to the lower yield point of the material Q235, the three samples deform elastically and the distributions of the grains change little microscopically. Therefore, the normalized ultrasonic energy diffusion coefficient fluctuates as the tensile force increases. When the tensile force is larger than 30 kN, the samples start to yield and the grain boundary sliding occurs. As a result, the boundary of the grains changes, and the normalized ultrasonic energy diffusion starts to decrease, which means the attenuating effect of ultrasonic energy is strengthened [61]. When the tensile force is about 40 kN, the dimension of the samples changes greatly due to large plastic deformation and necking effect, and it starts to have an influence on the normalized ultrasonic energy diffusion. Therefore, the normalized ultrasonic energy diffusion starts to increase again.

Both Figure 8 and Figure 9 indicate that when the tensile force is less than the lower yield point, the tensile force has a limited influence on the normalized ultrasonic energy diffusion coefficient, while when the load is larger than the lower yield point, the plastic deformation occurs in the sample, and the load starts to have a great influence on the coefficient.

### 4.3. Ultrasonic Energy Density-Based Tensile Force Identification

As we discuss in Section 4.1, the normalized ultrasonic energy diffusion coefficient shows some changing pattern as the tensile load increases. However, this changing pattern is not so obvious that it can be employed to monitor the tensile load, especially the force in the elastic deformation range. On the other hand, most of the metallic structures are only permitted to work in the elastic deformation range and deform elastically. Moreover, many structural damages are usually caused by the structural load. Therefore, the monitoring of the structural load in the elastic range will have potential engineering application value. For example, bolts are required to work in the elastic range and the looseness of the bolts is directly associated with the structural load, and therefore, the looseness can be detected by monitoring the load of the bolts.

Moreover, as addressed in Section 2, the ultrasonic energy density is sensitive to the microstructure of medium, and the microstructure including the structural damage (such as the looseness of bolts) is usually associated with structural load. Therefore, an ultrasonic energy density-based index is proposed to quantitatively monitor the tensile force and the tensile force index is given by

(4) $$I_{i}=\sqrt{\frac{\sum_{k=1}^{N}{\left(E_{i}(x,k\Delta t)-E_{0}(x,k\Delta t)\right)}^{2}}{\sum_{k=1}^{N}E_{0}^{2}(x,k\Delta t)}}$$

where $E_{i}(x,k\Delta t)$ is the ultrasonic energy density of the *k*th segment at the *i*th load step, $E_{0}(x,k\Delta t)$ is the ultrasonic energy density of the *k*th segment when there is no tensile force applied on the sample, and *N* is the total load steps.

**Figure 10:**
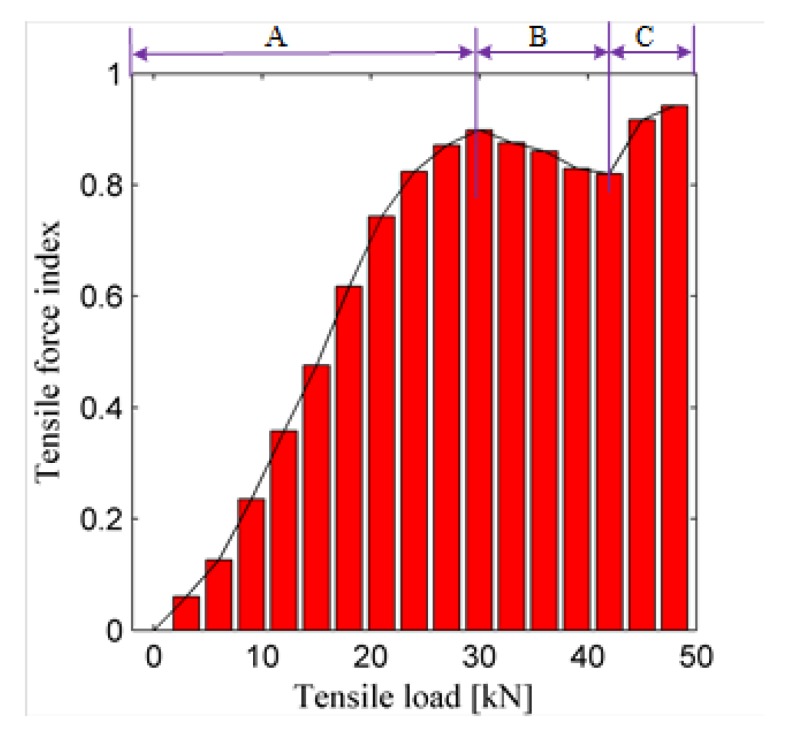
Tensile force index versus the tensile force for sample L1. **A**—Elastic deformation stage; **B**—Plastic deformation stage; **C**—Necking stage.

Figure 10 plots the force index of Sample L1 versus the tensile force. Figure 10 indicates that the proposed tensile force index shows a similar changing trend to the recorded displacement of Sample L1. The three deformation stages, including the elastic deformation, plastic deformation and necking stages, are observed in this curve. It also clearly shows that during the elastic deformation stage, i.e., when the tensile force is less than about 30 kN, the proposed load index does not fluctuate but increases linearly as the tensile force increases.

**Figure 11:**
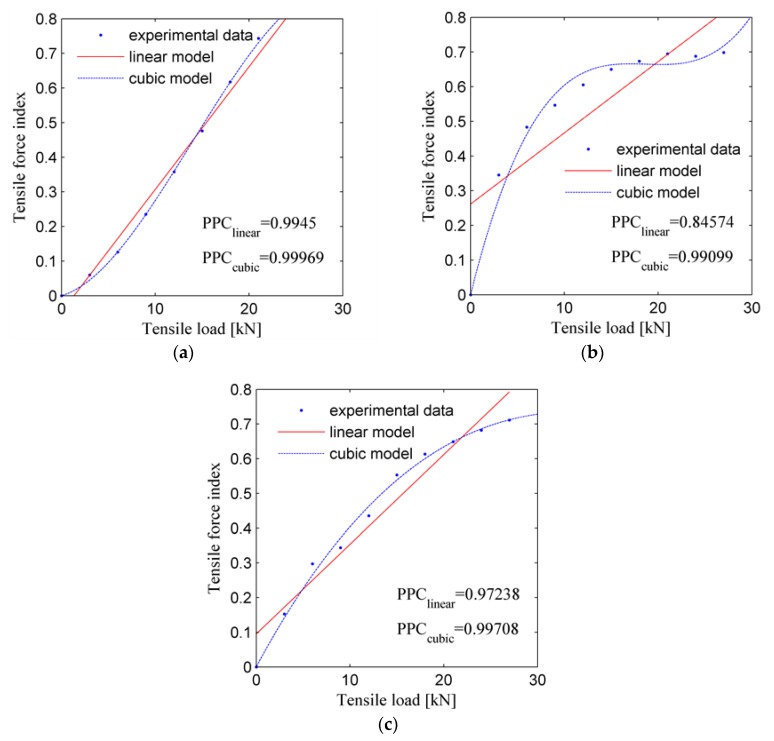
Tensile force index of the three samples during the elastic deformation stage: (**a**) Sample L1; (**b**) Sample L2; (**c**) Sample L3.

To further investigate the characteristics of the tensile force index during the elastic deformation stage, the tensile force indices of these three samples during the elastic deformation stage are plotted in Figure 11. Moreover, the tensile force index is approximated by a linear mode (solid line as shown in Figure 11), and the Pearson correlation coefficient (PCC) is also computed to measure the correlation between the approximated and experimental data. As shown in Figure 11, the value of PPC of Figure 11c is about 84.6%, while the value of the other two figures is more than 97.2%, which indicates that the tensile load index increases approximately linearly as the tensile force increases in the elastic deformation stage.

However, Figure 11b,c also indicate that the tensile force index starts to saturate when the tensile load is near to 20 kN. Therefore, a cubic model is also used to approximate the tensile force index. As shown in Figure 11, the PPCs of the cubic model are more than 99.1%, which indicates that the cubic model may be more suitable for approximating the tensile force index in the elastic deformation range.

Therefore, the proposed tensile force index can be used to monitor the tensile force in the elastic deformation stage and it will have a potential application of structural load-related damage detection.

It should also be noted that, due to the differences between the microstructures of the three samples, the fitted curves do not superimpose very well each other.

### 4.4. Discussions

Due to the change of the microstructure of the material in the loading process, the propagation of the ultrasound also changes and the normalized ultrasonic energy diffusion coefficient fluctuates firstly, then decreases, and at last increases as the tensile force increases. To better reflect the change of the ultrasonic energy diffusion, an ultrasonic energy density based tensile force index is proposed. The proposed tensile force index shows a similar changing trend to the recorded displacement of the sample during the loading process. Moreover, in the elastic deformation range, the proposed index firstly increases linearly as the tensile force increases, and then it saturates. A cubic model can be used to approximate the tensile force index in the elastic deformation range. Therefore, the proposed tensile force index can be used to monitor the tensile force in the elastic deformation range. In addition, based on the findings in this paper, in the future some force evaluation methods and their potential applications, such as the looseness detection of bolts, can be developed based on the linear relationships between the proposed index and the applied force.

## 5. Conclusions

The characteristics of ultrasonic energy diffusion in a steel alloy plate with different tensile forces are investigated in this paper by using piezoceramic transducers. Ultrasonic waves are usually attenuated by scattering at boundary between the grains during the propagation in a polycrystalline material, and the ultrasonic energy loss due to the scattering is described by the ultrasonic energy diffusion equation. In theory, when a force is applied onto a material, the material deforms macroscopically and the shape and boundaries of the grains distributed in the material also change microscopically. Therefore, the structural load has an influence on the ultrasonic energy diffusion during its propagation.

To investigate the influence of the structural load on ultrasonic energy diffusion, an experimental setup of a Q235 plate under different tensile forces is designed and four samples with similar dimensions are fabricated. The experimental results of four samples reveal that the proposed ultrasonic energy density based tensile force index and normalized ultrasonic energy diffusion coefficient changes as the tensile force increases. During the loading process, the normalized ultrasonic energy diffusion coefficient fluctuates firstly, then decreases and at last increase as the tensile force increases. To better reflect the change of the ultrasonic energy diffusion, an ultrasonic energy-density-based tensile force index is proposed. The proposed tensile force index shows a similar changing trend to the recorded displacement of the sample, and during the elastic stage, a cubic model can be used to approximate the proposed tensile force index. Therefore, the proposed tensile force index can be used to monitor the tensile force in the elastic deformation range.

Future work will involve the characterizations of ultrasonic energy diffusion under compressive force that varies in a non-monotonic fashion and the microscopic explanation for the change of ultrasonic energy diffusion under structural loads. It will involve the higher order polynomial or power approximation of the correlation between the tensile load and tensile load index. It will also involve force-related damage detection based on ultrasonic energy diffusion and its potential applications, such as the bolt looseness detection, in structural health monitoring.
